# Neuropathologic scales of cerebrovascular disease associated with diffusion changes on MRI

**DOI:** 10.1007/s00401-022-02465-w

**Published:** 2022-07-16

**Authors:** Aivi T. Nguyen, Naomi Kouri, Sydney A. Labuzan, Scott A. Przybelski, Timothy G. Lesnick, Sheelakumari Raghavan, Robert I. Reid, R. Ross Reichard, David S. Knopman, Ronald C. Petersen, Clifford R. Jack, Michelle M. Mielke, Dennis W. Dickson, Jonathan Graff-Radford, Melissa E. Murray, Prashanthi Vemuri

**Affiliations:** 1 Department of Laboratory Medicine and Pathology, Mayo Clinic, Rochester, MN USA; 2 Department of Neuroscience, Mayo Clinic, 4500 San Pablo Road, Jacksonville, FL 32224 USA; 3 Department of Quantitative Health Sciences, Mayo Clinic, Rochester, MN USA; 4 Department of Radiology, Mayo Clinic and Foundation, 200 First Street SW, Rochester, MN 55905 USA; 5 Department of Information Technology, Mayo Clinic, Rochester, MN USA; 6 Department of Neurology, Mayo Clinic, Rochester, MN USA

**Keywords:** Cerebrovascular disease, Neuropathology, Imaging, Diffusion MRI

## Abstract

Summarizing the multiplicity and heterogeneity of cerebrovascular disease (CVD) features into a single measure has been difficult in both neuropathology and imaging studies. The objective of this work was to evaluate the association between neuroimaging surrogates of CVD and two available neuropathologic CVD scales in those with both antemortem imaging CVD measures and postmortem CVD evaluation. Individuals in the Mayo Clinic Study of Aging with MRI scans within 5 years of death (*N* = 51) were included. Antemortem CVD measures were computed from diffusion MRI (dMRI), FLAIR, and T2* GRE imaging modalities and compared with postmortem neuropathologic findings using Kalaria and Strozyk Scales. Of all the neuroimaging measures, both regional and global dMRI measures were associated with Kalaria and Strozyk Scales (*p* < 0.05) and modestly correlated with global cognitive performance. The major conclusions from this study were: (i) microstructural white matter injury measurements using dMRI may be meaningful surrogates of neuropathologic CVD scales, because they aid in capturing diffuse (and early) changes to white matter and secondary neurodegeneration due to lesions; (ii) vacuolation in the corpus callosum may be associated with white matter changes measured on antemortem dMRI imaging; (iii) Alzheimer’s disease neuropathologic change did not associate with neuropathologic CVD scales; and (iv) future work should be focused on developing better quantitative measures utilizing dMRI to optimally assess CVD-related neuropathologic changes.

**Supplementary Information:**

The online version contains supplementary material available at 10.1007/s00401-022-02465-w.

## Background

Cerebrovascular disease (CVD) is an important contributor to cognitive decline and dementia in the elderly [2, 29]. CVD is a multifactorial process thought to encompass atherosclerosis, arteriolosclerosis, and cerebral amyloid angiopathy, while mechanisms underlying small vessel disease (SVD) include blood–brain barrier dysfunction, impaired vasodilation, vessel stiffening, and dysfunctional interstitial drainage and flow [36]. While a single measure to capture CVD would have significant clinical value, summarizing the multiplicity and heterogeneity of CVD features into a single number has been difficult in both neuropathology and imaging studies.

CVD is a broad term, and, as such, neuropathologic consensus and standardization for neuropathologic evaluation in dementia cases is continually evolving. Two available neuropathologic parameters to evaluate CVD of an atherosclerotic or arteriolosclerotic type are (1) Strozyk scale [32], which is based on presence and number of three macroscopic lesions (large infarctions, lacunar infarctions, and leukoencephalopathy) and (2) Kalaria scale [6, 11] which provides a more detailed evaluation of vessel wall modifications (arteriolosclerosis, angiopathy), perivascular spaces, myelin loss, microinfarcts, and large infarcts.

Measurement of imaging CVD features using MRI has been more standardized [36]. The clinical impact of MRI CVD features has been well studied; FLAIR and T2*GRE or SWI are commonly used neuroimaging MRI methods to assess CVD in aging and dementia studies [10]. FLAIR provides information about white matter hyperintensities and infarctions and T2* GRE/SWI provides information about microbleeds. Recent work has shown that dMRI may be an earlier indicator of CVD-related damage and may be a clinically useful CVD biomarker [1, 9, 20, 21, 35]. Two CVD markers from diffusion MRI (dMRI) were included in this study. First, a regional measure based on dMRI signal from genu of the corpus callosum which was associated with prodromal cerebrovascular health and was a strong predictor of cognitive performance in comparison to the visible lesions from FLAIR and T2* GRE images [34]. Second, a global measure called peak width of skeletonized mean diffusivity (PSMD), which is increasingly being used as a small vessel disease marker across studies [9].

Given this potential early biomarker for evaluating white matter changes that may contribute to overall cerebrovascular health, we hypothesize that neuroimaging parameters, including dMRI, may map to neuropathologic changes seen on postmortem and may more readily capture white matter changes. Thus, the overall objective of this work was to evaluate the association between neuroimaging surrogates of CVD and two available neuropathologic CVD scales in those with both antemortem imaging CVD measures and postmortem CVD evaluation. A secondary goal was to understand the neuropathologic underpinnings of the white matter changes observed in the anterior corpus callosum, which has been a prominent area of change related to systemic vascular disease.

## Materials and methods

### Selection of participants and demographics

The Mayo Clinic Study of Aging (MCSA) is a population-based study of Olmsted County, MN where residents were enumerated using the Rochester Epidemiology Project (REP) medical record-linkage system [27, 28, 30, 31]. We identified *N* = 51 individuals in Mayo Clinic Study of Aging (mean age of 83.8 years, 37% female) with dMRI scans within 5 years of death (Table 1). The MCSA design and clinical diagnoses criteria were discussed in detail by Petersen et al. [26] and Roberts et al. [15].

**Table 1:** Characteristics table with the mean (SD) listed for the continuous variables and count (%) for the categorical variables

	< 5 year *N* = 51	< 3 year *N* = 31
Male, no. (%)	32 (63%)	20 (65%)
Age, yrs	84 (7.5)	84 (7.7)
APOE4 carrier, no. (%)	16 (31%)	12 (39%)
Education	15 (3.2)	15 (3.3)
Global cognitive z-score	− 0.98 (1.43)	− 0.99 (1.52)
Time to death, years	2.5 (1.2)	1.6 (0.7)
Kalaria score	3.8 (2.2)	3.9 (2.2)
Strozyk score	1.2 (1.2)	1.3 (1.3)

Individuals in Mayo Clinic Study of Aging with MRI scans within 5 years of death (left column) and 3 years of death (right column) with available dMRI scans.

### Neuroimaging measures

All MRI images were acquired on 3 T MRI systems (GE Healthcare, Chicago, IL). CVD assessments: The CVD image assessments on FLAIR, T2* GRE, and dMRI were used in this study and shown on study participants in Table 1. FLAIR images were used to compute white matter hyperintensities [WMH] and total number of infarctions and T2* GRE were used to compute total number of microbleeds as previously published [15].

dMRI measures: dMRI images were acquired with 2.7 mm isotropic resolution spin echo axial Echo Planar Imaging sequence with five *b* = 0 followed by 41 *b* = 1000 s/mm^2^ diffusion weighted volumes. We used labels from the JHU “Eve” WM atlas [25] processed as previously described [35]. We computed fractional anisotropy of the genu of the corpus callosum [FAGCC] and fractional anisotropy of cingulum adjoining the hippocampus [FACGH] from the JHU atlas. We considered FAGCC as a white matter CVD marker due to its sensitivity to systemic vascular health [35]. There is greater myelin loss in the frontal, as opposed to temporal lobes, due to vascular disease [14, 17]. We also included FACGH as a control regional measure from dMRI, because it is impacted by tau pathology. This regional comparison difference between FACGH and FAGCC will facilitate contrasting impact of primarily vascular disease versus mixed etiologies on FACGH. We also included the global measure of white matter microstructure integrity in an unbiased anatomic fashion, peak width of skeletonized mean diffusivity (PSMD), which is commonly used and was calculated using the latest software [1].

**Fig. 1:**
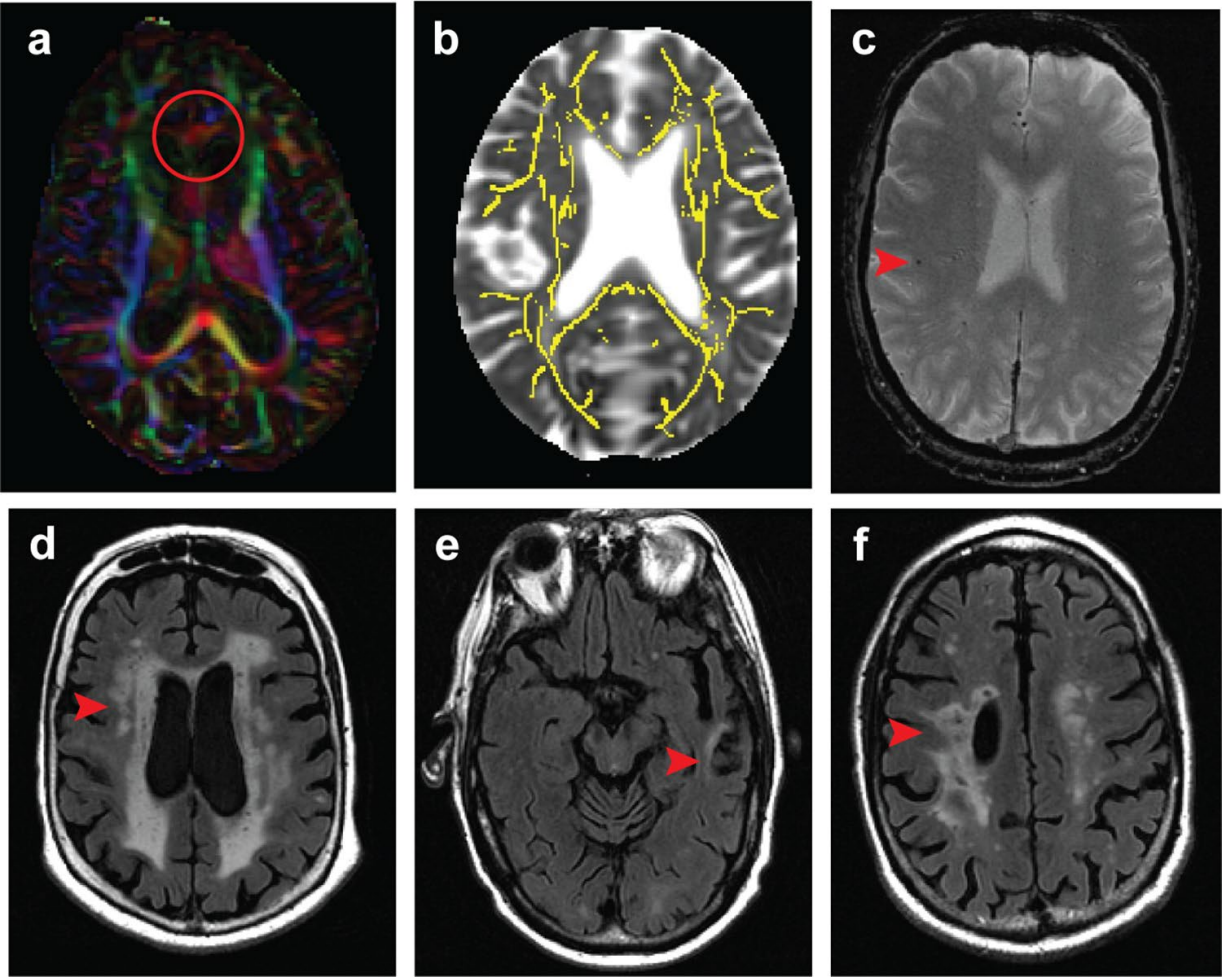
Measured CVD-related changes (**a**) dMRI seen as a fractional anisotropy map color map demonstrating changes in the genu of the corpus callosum measures as dMRI marker; (**b**) mean diffusivity image with peak skeletonized median diffusivity overlaid in red to yellow color scale; (c) T2-weighted MRI show cerebral microbleeds; (**d**) white matter intensities on FLAIR MRI. Both (**e**) cortical infarcts and (**f**) subcortical infarcts seen on FLAIR are demonstrated here

Summary CVD scores: Though not the focus of this manuscript, we also made comparisons with summary scores as sensitivity analyses. We compared two summary CVD scales. First, we computed a score in our data using methods proposed by Staals et al. [36] that summarizes all small vessel disease measures into one measure based on latent variable modeling. For this purpose, we computed periventricular and deep WMH were rated on the Fazekas scale (each 0–3) using FLAIR images [13]. Because T2-weighted images were not part of the imaging protocol, we did not include perivascular spaces in the modified Staals et al. summary score and included total number of infarcts instead of lacunes alone. The computed factor analysis provided the summation of 11.59*periventricular WMH + 9.15* deep WMH +0.36*infarctions +0.46*microbleeds for the modified Staals score. Second, our recently developed imaging CVD composite score which summarizes the variability in all individual CVD imaging measures described above (Fig. 1) [34]. This CVD composite score uses WMH as a quantitative measure instead of manual assessment based on Fazekas used in the Staals score. The CVD summary score was calculated from the simple linear summation of the principle components (retaining the scaling through principle component analyses) of white matter hyperintensities, FAGCC, cerebral microbleeds, and infarcts found on T2*GRE, FLAIR, and dMRI [34].

### Neuropathologic assessment and immunohistochemistry

Neuropathologic sampling followed recommendations of the Consortium to Establish a Registry for Alzheimer’s disease (CERAD) [22]. Single immunohistochemistry (IHC) was performed on 5 µm formalin-fixed, paraffin-embedded tissue sections. Briefly, serial 5 µm thick formalin-fixed paraffin-embedded brain sections were immunostained on a Thermo Fisher Lab Vision 480S autostainer using 3,3-diaminobenzidine (DAB) as the chromogen.

For neuropathologic diagnosis, antibodies against beta-amyloid, phospho-tau, and phospho-TDP-43 were used (Table S1). To evaluate white matter myelination, axonal density, and degradation, immunohistochemical evaluation was performed using antibodies to detect myelin basic protein (MBP) and SMI31 to assess axonal density (Table S1). The sections were counterstained with hematoxylin.

### Neuropathologic CVD scales

**Fig. 2:**
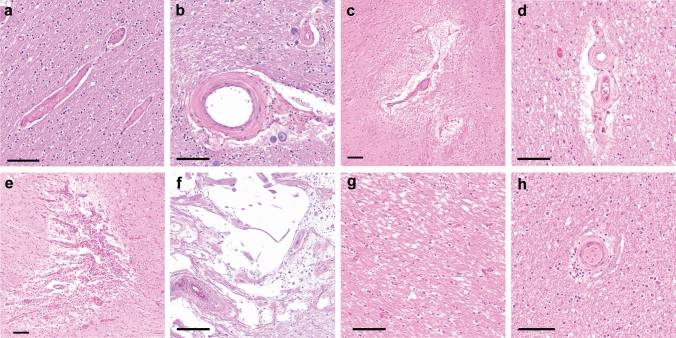
Neuropathologic findings, including evaluation by both Kalaria and Strozyk CVD scales and white matter abnormalities. **a** Histologically normal small caliber vessel, compared to **b** arteriolosclerosis, **c** concomitant perivascular white matter rarefaction, **d** focal perivascular hemosiderin deposition, **e** microinfarct, and **f** macroinfarct. **g** Extent of white matter vacuolation in the anterior corpus callosum and **h** arteriolosclerosis present within corpus callosum. Images white balanced. Scale bar = 100 um

Both the Kalaria and Strozyk scales were used to retrospectively evaluate neuropathologic severity of CVD documented in neuropathology reports. Briefly, the modified Kalaria scale was used to score arteriolosclerosis, cerebral amyloid angiopathy, perivascular tissue rarefaction, perivascular hemosiderin deposition, microscopic infarcts, and large infarcts on both neocortical and basal ganglia sections (Fig. 2).

Kalaria scale: hematoxylin and eosin-stained sections were reviewed at the time of neuropathologic evaluation. Abstraction of key information from neocortical regions (0–6 points) and basal ganglia (0–4 points) were used to evaluate CVD severity for a total of 10 points on the modified Kalaria scale [6, 11, 37], as previously described. Of note, the Kalaria scale evaluates vessel wall modifications (arteriolosclerosis, amyloid angiopathy), perivascular and white matter modification, cortical microinfarcts, and cortical large infarcts (Table S2).

Strozyk scale: hematoxylin and eosin-stained sections were evaluated utilizing the Strozyk summary [32], which is based upon three macroscopic vascular lesions: large infarcts (0–2), lacunar or cystic infarcts (0–2), and leukoencephalopathy (0–2). The respective scores were assigned, and a total Strozyk score was generated (0–6) (Table S3).

### Digital pathology

Digital pathology methods using Leica’s Aperio technology were processed as previously described [7, 16, 18, 23, 24]. The corpus callosum was annotated on digitized slides and then analyzed using eSlide Manager. Positive pixel count macros were used to quantify % positivity for MBP and SMI31.

Vacuolation in the corpus callosum was measured on sections stained with hematoxylin and eosin. Once regions of interest were annotated, a positive pixel count macro was used and negative space % was deemed a surrogate for vacuolation.

### Statistical analyses

Weighted linear regression models with adjustments for MRI scan time to death were used to evaluate the associations between the antemortem neuroimaging measures and neuropathology scales. The results are discussed with the following notation—(estimate [SE], *p* value). The weighting was 1/time from MRI scan to death, so that those with MRI scans closer to death received more weight versus those farther away. The analyses included 51 study participants with scans within 5 years of death and a sensitivity analysis was done in 31 study participants with scans within 3 years of death (*N* = 31) (Table 1). Partial Pearson correlations were used to calculate neuroimaging parameters as a function of cognition and were adjusted for age, sex, education, and cycle number. One-way ANOVA analyses were performed with the vascular neuropathology variables where the groups were classified by their Alzheimer’s disease neuropathologic change (ADNC).

## Results

### Sample demographics and neuropathologic characteristics

Among the 51 autopsy cases, the mean age of death was 83.8 (± 7.5 years) and 19 were female (37%) (Table 1). Eleven cases showed low ADNC (22%), 12 cases were intermediate ADNC (24%), and 8 cases were high ADNC (16%) (Table S4). Of the total cases with ADNC, 13 showed concomitant Lewy bodies or Lewy body disease (LBD). Furthermore, the demographics between the autopsied and non-autopsied cases did not significantly differ by age, sex, APOE status, or global cognition but did differ in education level (Table S5).

### Neuropathologic scale results

**Fig. 3:**
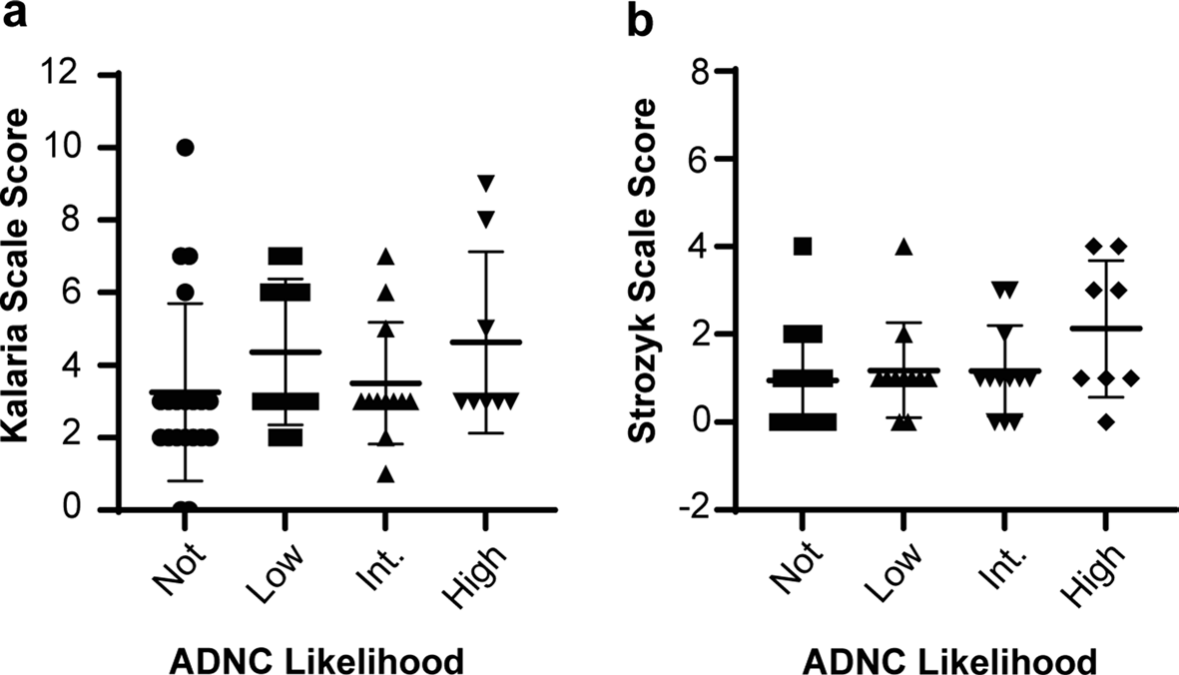
Distribution of the neuropathologic CVD scales as a function of ADNC likelihood: **a** Kalaria scale scores **b** Strozyk scale scores. Ordinary one-way ANOVA showed n.s. between groups

The average Kalaria score was 3.8 (S.D. 2.2), and the highest scores were observed in cases with either high ADNC or without ADNC (“Not” likelihood) (Fig. 3; Table S4). The average Strozyk score was 1.2 (S.D. 1.2). In contrast to the Kalaria score, the highest Strozyk scores were observed for cases with varying levels of ADNC. Lastly, ADNC did not associate with either neuropathologic CVD scales, evaluated by graphing the distribution of Kalaria or Strozyk scale scores as a function of ADNC likelihood (Fig. 3).

### dMRI parameters are best predictors of Kalaria scale

Of the neuroimaging variables, FAGCC (− 13.45 [6.14], *p* = 0.033) and FACGH (− 19.52 [9.01], *p* = 0.035) best predicted neuropath findings via Kalaria scale when the analyses was conducted on data within 5 years. The percent variability explained in Kalaria scale by dMRI models was about 17% in the analyses of scans within 5 years and 26% in the analyses of scans within 3 years (Table 2). Surprisingly, neither the CVD features from FLAIR nor T2* GRE explained a significant proportion of variability in the Kalaria scale. In the sensitivity analyses with 3 years between MRI scan and death, all the associations remained similar (Table 2). Of note, these dMRI metrics were relatively stable as a function of increasing time (slow trend of decrease in FA and slow trend of increase in PSMD as expected as a function of age), as demonstrated by longitudinal spaghetti plots (Figure S1).

**Table 2:** Linear regression results with an adjustment for time from scan to death and weighted for time

	5 years between scan and death (*N* = 51)				3 years between scan and death (*N* = 31)				
	Estimate (SE)	*P* value	*R*^2^	Partial R^2^	Estimate (SE)		*P* Value	*R*^2^	Partial *R*^2^
Imaging predicting Kalaria scale									
FAGCC	**− 13.45 (6.14)**	**0.033**	**0.1691**	**0.091**		− **16.83 (7.64)**	**0.036**	**0.2587**	**0.148**
FACGH	**− 19.52 (9.01)**	**0.035**	**0.1676**	**0.089**		**− 22.91 (10.90)**	**0.045**	**0.2487**	**0.136**
PSMD	5281 (2967)	0.081	0.1427	0.062		5259 (3551)	0.15	0.1934	0.073
WMH	0.12 (0.42)	0.78	0.0718	0.002		0.23 (0.52)	0.66	0.1242	0.007
Microbleeds	0.29 (0.25)	0.24	0.1072	0.031		0.24 (0.30)	0.43	0.1452	0.023
Infarcts	− 0.19 (0.29)	0.51	0.0776	0.009		− 0.13 (0.46)	0.78	0.1157	0.003
Imaging predicting Strozyk scale									
FAGCC	− **11.24 (3.41)**	**0.002**	**0.2311**	**0.184**	− **13.85 (4.34)**		**0.003**	**0.3396**	**0.267**
FACGH	− 9.2 (5.37)	0.093	0.1118	0.058	− 9.91 (6.93)		0.16	0.1608	0.068
PSMD	**5727 (1594)**	** < 0.001**	**0.2572**	**0.212**	**5719 (1982)**		**0.007**	**0.3059**	**0.229**
WMH	0.44 (0.23)	0.062	0.1107	0.072	0.48 (0.30)		0.12	0.1673	0.088
Microbleeds	0.16 (0.14)	0.27	0.075	0.028	0.18 (0.18)		0.33	0.1296	0.036
Infarcts	− **0.35 (0.16)**	**0.034**	**0.1314**	**0.078**	− 0.42 (0.27)		0.13	0.1643	0.087

The measures with *P* < 0.05 are indicated in bold

*FAGCC* Fractional anisotropy of genu of the corpus callosum, *CGH* Fractional anisotropy of cingulum adjoining the hippocampus, *PSMD* Peak width of Skeletonized Mean Diffusivity, *WMH* white matter hyperintensities, *CMB* cerebral microbleeds

### FAGCC and infarcts predict Strozyk scale

FAGCC was the key predictor of Strozyk scale (− 11.24 [3.41], *p* = 0.002), explaining 23% of the variability when analyzing scans within 5 years and 34% within 3 years of death (Table 2). Additionally, infarcts and Strozyk scale were also significantly associated. About 13% variability in the Strozyk scale was captured by infarcts when examining scans within 5 years of death.

### FAGCC associated with specific neuropathologic features

Among the three neuropathological features evaluated (Vacuolation, MBP, SMI31), only corpus callosum vacuolation was associated with FAGCC (*r* = − 0.32, *p* = 0.024) but not MBP (*r* = 0.08, 0.59) and SMI31 (*r* = 0.17, *r* = 0.24). However, after the weighted adjustment for time, this association between corpus callosum vacuolation and FAGCC was non-significant (*r* = − 0.28, *p* = 0.055).

### Imaging findings and global cognition

To evaluate how neuroimaging parameters associated with cognition levels, Partial Pearson Correlations were calculated between global cognition scores and dMRI specific imaging variables, including FAGCC, FACGH, PMSD, WMH, cerebral microbleeds, and infarcts (Table 3). FAGCC and PMSD were both significantly associated with global cognition, when adjusted for age, sex, education, and cycle number (Table 3).

**Table 3:** Partial Pearson correlations between global cognition score and neuroimaging parameters

	5 years between scan and death (*N* = 51)	
	Partial Pearson correlation	*P* value
Neuroimaging parameters		
FAGCC	**0.358**	**0.029**
FACGH	0.131	0.44
PMSD	− **0.318**	**0.055**
Log WMH	− 0.18	0.29
CMB	− 0.292	0.10
Infarcts	0.11	0.53

The measures with *P* < 0.05 are indicated in bold

*FAGCC* Fractional anisotropy of genu of the corpus callosum, *CGH* Fractional anisotropy of cingulum adjoining the hippocampus, *WMH* white matter hyperintensities, *CMB* cerebral microbleeds, *CVD* cerebrovascular disease

### Comparison with summary CVD scores

The results for the summary scores are shown in Supplemental Table S6. While neither the CVD composite nor the Staal score were predictors of Kalaria scale, the modified Staals score was predictive of the Strozyk score (0.03 [0.01], *p* = 0.001). However, dMRI measures presented in the paper outperformed the summary CVD scores supporting the main findings of the paper that dMRI may be a good surrogate of neuropathologic CVD scales, because it captures diffuse (and early) changes to white matter and secondary neurodegeneration due to lesions.

## Discussion

We evaluated neuroimaging CVD measures in the context of neuropathologic scales and found that: (i) Microstructural white matter injury measurement using dMRI was a useful surrogate of neuropathologic CVD scales and may aid in capturing diffuse (and early) changes to white matter and secondary neurodegeneration due to lesions; (ii) vacuolation in the corpus callosum may be associated with white matter changes on antemortem dMRI imaging; (iii) developing better quantitative measures leveraging these results are important in more accurately capturing CVD-related pathological changes.

In the recent past, extensive research has focused on improved neuroimaging CVD markers for aging and dementia. While information from FLAIR and T2* GRE/SWI MRI scans provide quantification of visible CVD lesions, early CVD changes (before appearance of lesions) have significant clinical diagnostic and prognostic value. There is increasing evidence that dMRI markers can capture these prodromal changes [1, 8, 20, 35]. Though we did not hypothesize that a specific marker would be more strongly associated with Kalaria and Strozyk scales, we found that dMRI markers had greater predictive power compared to FLAIR and T2* GRE markers. These results confirm that dMRI measures are good surrogates of microvascular and macrovascular brain changes and white matter changes are a key component of CVD changes seen in the brain. Both FAGCC and FACGH significantly predicted Kalaria scale but only FAGCC predicted Strozyk scale. A reason for this difference could be the consideration of microvascular disease in Kalaria scale which captures any microvascular disease independent of etiology. This contrasts with the Strozyk scale which has a greater focus on macrovascular disease which generally has a greater impact on frontal lobes. Lastly, dMRI markers, FAGCC and PSMD were not only useful in neuropathologic evaluation but were also strongly associated with global cognition, underscoring that these vascular imaging markers may be independent drivers of cognition, although this finding requires further investigation.

Microbleeds, infarcts, and WMH were not the strongest predictors of either scale. There is considerable heterogeneity in the location of these markers and the summation of these lesions across the brain on antemortem imaging may not map onto CVD seen on a sampled portion of the brain for postmortem evaluation. Contrasting the performance of dMRI measurements with FLAIR and T2* GRE measurements supports the point that white matter microstructural integrity may be key for capturing CVD for clinical and research studies. Currently, areas sampled for autopsy studies are designed to capture neurodegenerative changes rather than map onto areas where a high burden of cerebrovascular disease may occur, i.e., vascular territories, which is why a more global measure of white matter maps onto the neuropathology scales better than focal lesions (i.e., infarcts do not explain significant variability in the CVD neuropathologic scales).

Another key finding in this study was the modest association of dMRI with white matter vacuolation seen in the corpus callosum. Although white matter injury is frequently described in aging and neurodegeneration, the mechanism remains unclear, and inciting etiologies can be multifactorial. Various animal model studies have shown an underlying etiology of intramyelinic edema secondary to ischemia and hypoperfusion in contributing to white matter vacuolation, evaluated histologically [12]. Of note, a histologically similar pattern may also be seen in the setting of axonal injury, which may be secondary to several etiologies, including neurodegeneration, diffuse axonal injury, infection, amongst others [4]. Broadly, beyond the corpus callosum, white matter vacuolation secondary to intramyelinic edema has been seen in mitochondrial encephalopathies, particularly Kearns-Sayre syndrome, neurodegenerative leukoencephalopathies, vitamin B12 deficiency, and various toxin-related leukoencephalopathies [3, 19, 33]. Overall, dMRI association with histologic white matter change may support FAGCC as a parameter in evaluating for early white matter CVD changes, particularly the corpus callosum, in antemortem cases. The primary signal coming from vacuolation suggests that free water measures from multi-compartment modeling of white matter microstructure using Neurite Orientation Dispersion and Density Imaging (NODDI) may be a more sensitive marker of early CVD changes, although the underlying mechanism and direct relationship to CVD requires further study. Of note, while not the primary aim for this study, when neuropathologic CVD scales were compared to ADNC likelihood, there were no strong associations between the two (Fig. 3). While the associations between these etiologies are highly debated, there may be low degree of association at a more general level [5].

Though not the focus of this manuscript, sensitivity analyses were also performed using modified Staals score [35] and composite CVD marker [34] that we developed as a summation of all the neuroimaging markers discussed here. The data are shown in the supplemental material (Table S6). We found that dMRI outperformed these summary scores, because these were not specifically designed (or weighted) to map onto postmortem evaluation.

Limitations to the study, however, are that we only used a few parameters, such as 2D T2* GRE and 2D FLAIR images for lesion assessments. 3D scans may have greater sensitivity to lesion detection and quantification. At the time of evaluation, antemortem multi-shell dMRI and 3D sequences for lesion quantification on those with postmortem evaluation were not available. For this study, we considered a small number of CVD markers due to their ready availability, and additional data acquisition and processing imaging methods may provide markers that have higher predictive value for neuropathologic outcomes.

To date, few studies have shown the relationships between antemortem neuroimaging CVD parameters with postmortem neuropathologic evaluation. Herein, we demonstrated that specific dMRI measurements predicted key vascular features seen in neuropathologic evaluation. Given the significance of CVD in aging and dementia, the development of valid, predictive quantitative neuroimaging measures that capture CVD neuropathology, using newly emerging technologies such as NODDI, will be of significant interest in future research studies.

## Supplementary Information

Below is the link to the electronic supplementary material.

Supplementary file1 (PDF 454 KB)
