# Supplementary material for: Neuropathologic scales of cerebrovascular disease associated with diffusion changes on MRI
Source: Acta Neuropathol. 2022 Jul 16;144(6):1117–25. doi: 10.1007/s00401-022-02465-w (PMC9637622; doi:10.1007/s00401-022-02465-w)

Title: Neuropathologic Scales of Cerebrovascular Disease Associated with Diffusion Changes on MRI

Authors: Aivi T. Nguyen[1], Naomi Kouri[2], Sydney A. Labuzan[2], Scott A. Przybelski[3], Timothy G. Lesnick[3], Sheelakumari Raghavan[4], Robert I. Reid[5], R. Ross Reichard[1], David S. Knopman[6], Ronald C. Petersen[6], Clifford R. Jack Jr.[4], Michelle M. Mielke[3], Dennis W. Dickson[2], Jonathan Graff-Radford[6], Melissa E. Murray[2], Prashanthi Vemuri[4]

Affiliations: [1]Department of Laboratory Medicine and Pathology, Mayo Clinic, Rochester, MN; [2]Translational Neuropathology Laboratory, Mayo Clinic, Jacksonville, FL; Departments of [3]Quantitative Health Sciences, [4]Radiology, [5]Information Technology, and [6]Neurology Mayo Clinic, Rochester, MN

Corresponding Author(s):

Melissa E. Murray, PhD
Translational Neuropathology Laboratory
Mayo Clinic Florida
4500 San Pablo Road
Jacksonville, Florida 32224
Phone: 904-953-1083 E-mail: murray.melissa@mayo.edu

Prashanthi Vemuri, Ph.D.
Mayo Clinic and Foundation
200 First Street SW, Rochester, MN 55905
Phone: +1 507 538 0761, E-mail: vemuri.prashanthi@mayo.edu

Table S1. Antibodies used in immunohistochemical staining.

Antibody Name	Clone	Concentration	Host	Source	Catalog #
Anti-Beta-amyloid	Monoclonal (6F/3D)	1:100	Mouse	DAKO	M0872
Anti-phosphoPHF-tau	Monoclonal (AT8)	1:100	Mouse	ThermoFisher	MN1020
Anti-phosphoTDP-43	Monoclonal (pS409/410)	1:10,000	Mouse	Cosmo Bio	TIP-PTD-M01
Anti-Myelin Basic Protein	Monoclonal (119-131, clone 2)	1:500	Mouse	Millipore/Sigma	MAB381
Anti-Neurofilament, Phosphorylated	Monoclonal (SMI-31)	1:40,000	Mouse	Covance	SMI-31R

Table S2. Modified Kalaria CVD Score Rubric

**Kalaria cerebrovascular disease scale [5-6]**	
**Cerebral Cortex** (select highest that applies)	
☐ **0** Normal appearance of brain, vessels, white matter, and cortex	
☐ **1** Mild modification of vessel walls, perivascular spaces, or white matter	
☐ **2** Moderate to severe but isolated modification of the vessel (arteriolosclerosis or amyloid angiopathy), usually associated with hemosiderin deposits in the perivascular spaces; and/or Moderate to severe cerebral amyloid angiopathy involving parenchyma	
☐ **3** Moderate to severe perivascular space dilatations either in the deep or the juxtacortical white matter	
☐ **4** Moderate to severe myelin loss; and/or White matter infarct	
☐ **5** Presence of cortical microinfarcts	
☐ **6** Presence of large infarcts and/or cystic infarcts	
**Basal ganglia** (select highest that applies)	
☐ **0** Normal appearance	
☐ **1** Mild modification of vessel walls or perivascular spaces<br>(or if PVS not noted, but isolated moderate to severe arteriolosclerosis)	
☐ **2** Moderate to severe perivascular space dilatations	
☐ **3** Presence of microinfarcts	
☐ **4** Presence of large infarcts; and/or lacunar infarct	
**Kalaria score** (Total of cortex and basal ganglia)	(out of 10)

Table S3. Strozyk CVD Score Rubric

**Strozyk Scale Rubric**	
**Large infarct**	
None	0
1 large infarct	1
≥2 large infarcts	2
**Lacunar/Cystic infarcts**	
None	0
1 lacunar infarct	1
≥2 large infarcts	2
**Leukoencephalopathy**	
None	0
Mild	1
Moderate-to-Severe	2
**Total vascular score**	(out of 6)

Table S4. Neuropathologic Characteristics of cases

Case Number	Sex	PMI (hours)	Braak Stage	Thal Phase	Neuropathologic Diagnosis(es)	Kalaria Score	Strozyk Score	*APOE ε4*
1	Male	19	IV	3	Intermediate ADNC	3	0	No
2	Male	11	II	4	Low ADNC	2	1	Yes
3	Male	14	III	3	Intermediate ADNC	3	1	No
4	Female	18	IV	5	Intermediate ADNC	5	2	Yes
5	Female	18	IV	1	No ADNC	6	1	No
6	Male	6	IV	2	No ADNC	3	1	No
7	Male	7	IV	5	Intermediate ADNC	6	1	No
8	Male	19	I	2	Low ADNC	6	2	No
9	Female	21	III	0	No ADNC	2	0	No
10	Female	3	V	5	High ADNC	3	0	No
11	Male	12	V	5	High ADNC	3	1	Yes
12	Male	4	III	3	Intermediate ADNC	3	1	No
13	Male	21	II	1	Low ADNC	6	0	Yes
14	Female	30	IV	2	Low ADNC	7	1	Yes
15	Male	23	III	0	No ADNC	7	2	No
16	Female	44	II	5	Low ADNC	2	0	Yes
17	Male	16	III	3	Intermediate ADNC	2	0	No
18	Male	7	IV	3	Intermediate ADNC	7	3	Yes
19	Male	21	II	1	Low ADNC	3	1	No
20	Female	46	II	4	Low ADNC	7	4	Yes
21	Male	18	III	1	No ADNC	2	0	No
22	Female	19	III	0	No ADNC	2	0	Yes
23	Male	11	III	5	Intermediate ADNC, Transitional LBD	3	1	Yes
24	Female	51	II	3	Low ADNC	3	1	No
25	Male	5	VI	5	High ADNC, Amygdala-predominant Lewy bodies	8	4	No
26	Male	5	III	2	No ADNC	0	2	No
27	Female	14	I	3	No ADNC	2	0	Yes
28	Female	21	III	3	No ADNC	3	1	No
29	Male	12	III	0	No ADNC	3	1	No
30	Male	13	V	3	Intermediate ADNC, Amygdala-predominant Lewy bodies	3	3	No
31	Male		II	5	Low ADNC, Transitional LBD	6	1	Yes
32	Male	27	II	1	No ADNC	2	0	No
33	Male	9	V	4	High ADNC	3	1	Yes
34	Female	15	III	0	No ADNC	10	4	No

35	Male	21	III	1	No ADNC, Transitional LBD	2	0	No
36	Male	4	IV	0	No ADNC, Transitional LBD	7	2	No
37	Male	24	IV	5	Intermediate ADNC	3	1	No
38	Female	46	II	2	Low ADNC	3	1	No
39	Male	22	III	1	No ADNC, Brainstem LBD	2	0	Yes
40	Female	45	VI	5	High ADNC, Diffuse LBD	3	3	No
41	Male	15	III	0	No ADNC	3	1	No
42	Male	20	I	0	No ADNC	0	0	No
43	Male	20	III	1	No ADNC	3	1	No
44	Female	6	III	3	Intermediate ADNC, Brainstem LBD	3	0	No
45	Male	18	IV	1	Low ADNC, Brainstem LBD	3	1	No
46	Female	15	V	5	High ADNC	5	4	No
47	Female	10	V	5	High ADNC, Diffuse LBD	3	1	Yes
48	Male	9	I	0	No ADNC	3	1	No
49	Female	44	V	5	High ADNC	9	3	Yes
50	Male	13	I	0	No ADNC	3	2	No
51	Female	12	III	1	Intermediate ADNC, Brainstem LBD	1	1	No

Abbreviations: PMI, postmortem interval; ADNC, Alzheimer's Disease Neuropathologic Change; LBD, Lewy Body Disease

Table S5. Characteristics table with the mean (SD) listed for the continuous variables and count (%) for the categorical variables.

	Not Autopsied<br>n = 843	Autopsied<br>n = 51	P-value
Male, no. (%)	448 (53%)	32 (63%)	0.18
Age, yrs	84.7 (7.6)	83.8 (7.5)	0.41
APOE ε4 carrier (%)	237 (29%)	16 (31%)	0.69
Education, yrs	13.5 (2.9)	15.0 (3.2)	<0.001
Global	-1.39 (1.36)	-0.98 (1.43)	0.064

P-values are from either a t-test or chi-squared test.

Table S6. Table of linear regression results with an adjustment for time from scan to death and weighted for time for the summary scores.

	5 years between scan and death (N=51)				3 years between scan and death (N=31)			
	Estimate (SE)	P-value	R^2	Partial R^2	Estimate (SE)	P-Value	R^2	Partial R^2
**Imaging Predicting Kalaria Scale**								
CVD Composite Score	0.07 (0.16)	0.67	0.0673	0.004	0.08 (0.21)	0.72	0.1174	0.005
Modified Staals Score	0.04 (0.019)	0.066	0.1347	0.076	0.04 (0.023)	0.081	0.2124	0.112
**Imaging Predicting Strozyk Scale**								
CVD Composite Score	-0.06 (0.09)	0.49	0.0449	0.011	-0.04 (0.13)	0.75	0.0889	0.004
Modified Staals Score	***0.03 (0.01)***	***0.001***	***0.2431***	***0.216***	***0.04 (0.013)***	***0.006***	***0.3208***	***0.258***

Figure S1. DTI parameter spaghetti plots, wherein serial lines are shown as a function of age

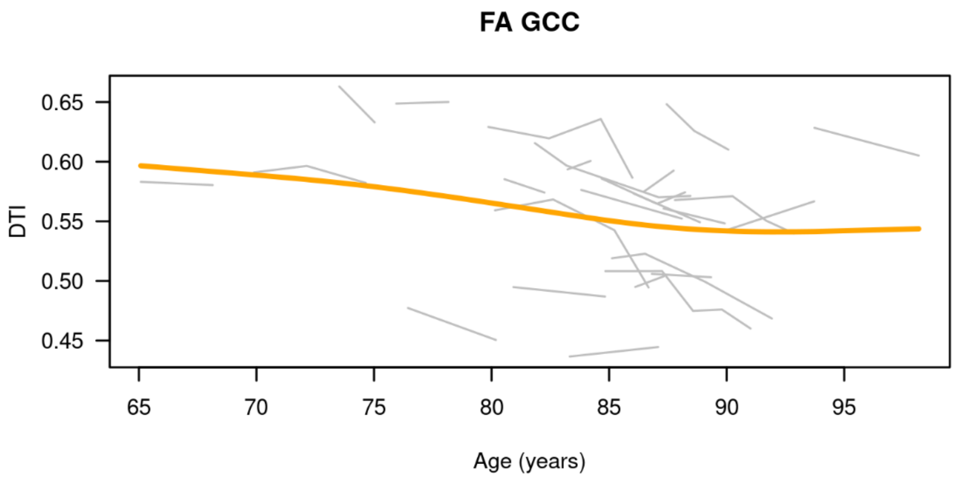

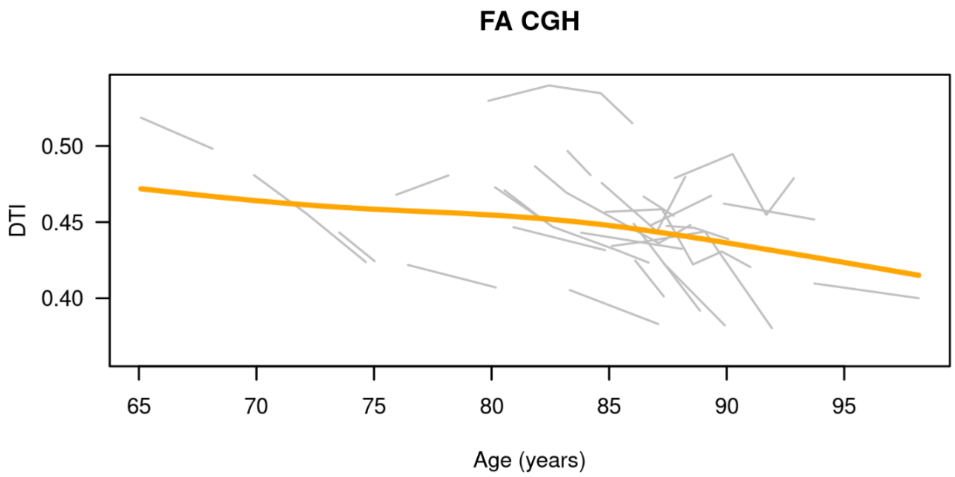

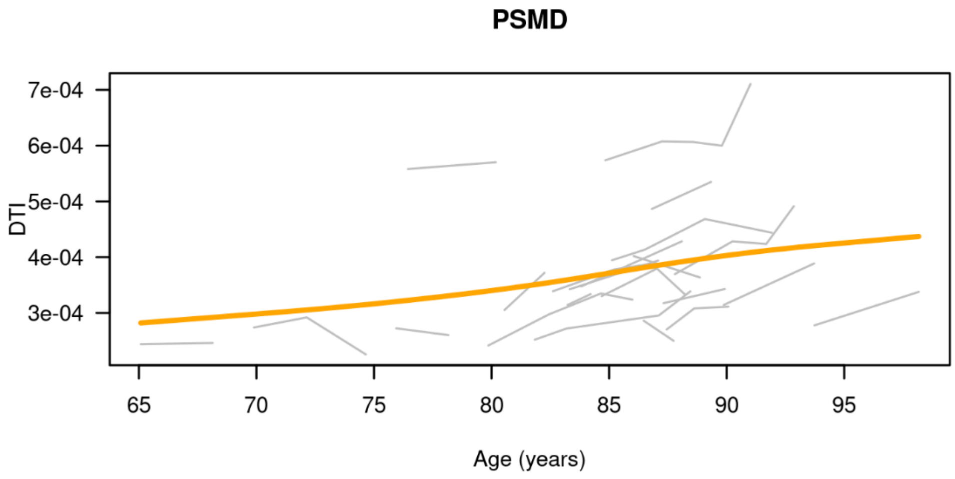

Supplement: Supplementary file 1 — Supplementary file1 (PDF 454 KB) [file 401_2022_2465_MOESM1_ESM.pdf]
